# Clinical Effects and Safety of Tongxieyaofang on Diarrhea Predominant Irritable Bowel Syndrome: A Meta-Analysis of Randomized Trails

**DOI:** 10.1155/2019/4893876

**Published:** 2019-01-06

**Authors:** Yuhong Zhou, Shutang Han, Yamin He

**Affiliations:** ^1^Nanjing University of Chinese Medicine, Nanjing, Jiangsu 210023, China; ^2^Department of Digestive endoscopy, Jiangsu Province Hospital of Traditional Chinese Medicine, Affiliated Hospital of Nanjing University of Chinese Medicine, Nanjing, Jiangsu 210029, China; ^3^Department of Pathology, Renji Hospital, School of Medicine, Shanghai Jiao Tong University, Shanghai 200001, China

## Abstract

*Background. *Tongxieyaofang (TXYF), a prescription originated from traditional Chinese medicine (TCM), has been widely used on treating Diarrhea Predominant Irritable Bowel Syndrome (IBS-D). The purpose of this meta-analysis was to investigate whether TXYF was effective and safe for IBS-D.* Methods.* We searched seven electronic databases including CENTRAL, MEDLINE, PubMed, CNKI, VIP, CBM, and Wanfang Data up to 26 July 2017. Randomized controlled trails (RCTs) were eligible, regardless of blinding. Risk of bias of included trials was evaluated according to the Cochrane Handbook.* Results. *The total number of participants analyzed in the meta-analysis was 3062, of which 1556 received TXYF, while 1506 received ordinary treatment. The primary outcome was clinical effective rate. Compared with conventional medication which included probiotics, pinaverium bromide, trimebutine, and Oryzanol, TXYF significantly improved the clinical effective rate (n=37, OR: 4.61; 95% CI: 3.67–5.78; P < 0.00001) and decreased the adverse events (n=10, OR: 0.26; 95% CI: 0.08–0.86; P = 0.03). There was not significant association with the score of abdominal pain, defecating frequency, fecal property, and total symptom.* Conclusions. *We suggested a moderate recommendation for TXYF on IBS-D, due to the fact that the risk of bias of the finally included trails was not high. Considering that all identified studies were not of high qualities and large samples, further rigorously designed and large scale RCTs were necessary to improve the applicability of our study results.

## 1. Introduction

Irritable bowel syndrome (IBS) is a chronic and sometimes disabling functional bowel disorder [1]. Worldwide, IBS negatively affects the quality of life and burdens the medical cost. The prevalence of IBS in China is between 6.53% to 15.02% [2, 3]. Rome diagnostic criteria and recommendations are commonly used in the design and performance of clinical researches in the field of IBS. Rome IV criteria, the current criteria for IBS, show that the diagnosis of IBS is abdominal pain at least 1 day per week during the last 3 months [4, 5]. The abdominal pain is associated with at least 2 of the following, defecation, change in stool frequency, and change in stool form [5]. On the basis of Rome IV criteria, IBS is divided into four subtypes based on symptoms, including IBS with prominent diarrhea (IBS-D), IBS with constipation (IBS-C), IBS with mixed symptoms of diarrhea and constipation (IBS-M), and untyped IBS (IBS-U) [5]. Traditionally, the pathogenesis of IBS was conceptualized as a brain-gut disorder because of its high association with central nervous system (CNS) alterations especially anxiety and depression [1]. Environmental factors including early life stressors, food intolerance, antibiotics, or enteric infection and host factors including altered pain perception, altered brain-gut interaction, dysbiosis, increased intestinal permeability, increased gut mucosal immune activation, or visceral hypersensitivity both contribute to IBS symptoms [6]. Due to the heterogeneity of IBS, it is difficult to design an algorithm to fit all patients [1]. Antidiarrheals, serotonin agents, and antispasmodics are often used as first-line or second-line agents in patients with IBS-D [6]. Medical treatments for IBS-C include fiber supplements, laxative agents, and prosecretory agents [6].

In China, Tongxieyaofang (TXYF) has been used in treating diarrhea for hundreds years. A system review (n=1125) shows that the effectiveness of TXYF is higher than the conventional medicine (risk ratio 1.35, 95% CI 1.21-1.50) in the management of IBS [7]. Therefore, our research is performed to investigate whether TXYF is effective and safe on the management of IBS-D.

## 2. Methods

### 2.1. Research Protocol

This meta-analysis was performed according to Preferred Reporting Items for Systematic Reviews and Meta-Analyses (PRISMA) statement. The protocol for this meta-analysis is available in PROSPERO (CRD42018105307).

### 2.2. Databases and Search Strategies

We searched seven electronic databases including CENTRAL, MEDLINE, PubMed, CNKI, VIP, CBM, and Wanfang Data up to 26 July 2017. The keywords were as follows (IBS-D or IBS-D∗) for IBS-D AND (tongxieyaofang or tongxieyaofang∗) for TXYF AND randomized or controlled or clinical research.

### 2.3. Eligibility Criteria

Studies were selected based on the following inclusion criteria: (a) any RCTs compared TXYF with ordinary treatment group or placebo, regardless of blinding; (b) no restriction on age, sex, country, or underlying diseases of participants; (c) trails provided records based on “the guiding principle of clinical research on new drugs of TCM” and/or “the diagnostic criteria of TCM syndrome”; (d) trails provided Rome diagnostic criteria.

### 2.4. Risk of Bias Assessment

According to the Cochrane Handbook for Systematic Reviews of Interventions (version 5.0.2), two independent researchers (Y. H. Zhou and S. T. Han) assessed the included trails independently in seven domains, included the randomization sequence generation, allocation concealment, blinding of participants and personnel, blinding of outcome assessment, incomplete outcome data, selective reporting, and other biases. The included trials were graded as low quality, high quality, or moderate quality based on the following criteria: (a) trails with either randomization or allocation concealment assessed as a high risk of bias were considered low quality; (b) trials with both randomization and allocation concealment assessed as a low risk and all other items assessed as low or unclear risk of bias were considered high quality; (c) trials were considered moderate quality if they did not meet criteria for high or low risk.

### 2.5. Study Selection and Data Extraction

Two researchers (Y. H. Zhou and S. T. Han) independently screened the titles and abstracts of the included trails and confirmed whether the trails meet the inclusion criteria. Two researchers above independently extracted the following information from each trail: the first author, year of publication, country of origin, sample size, participants (mean age and IBS-D duration), details of control inventions, treatment duration, and outcome measurements.

### 2.6. Statistical Analysis

Heterogeneity was evaluated by chi-square test. We performed meta-analysis to calculate risk ratios (RRs), absolute risk differences (ARDs), and 95% CIs using the Mantel-Haenszel statistical method. A random-effects model was used to pool the data, and statistical heterogeneity between summary data was evaluated using the* I*^2^ statistic. Statistical significant difference was considered as p-value < 0.5.

## 3. Results

### 3.1. Studies Retrieved and Characteristics

**Figure 1:**
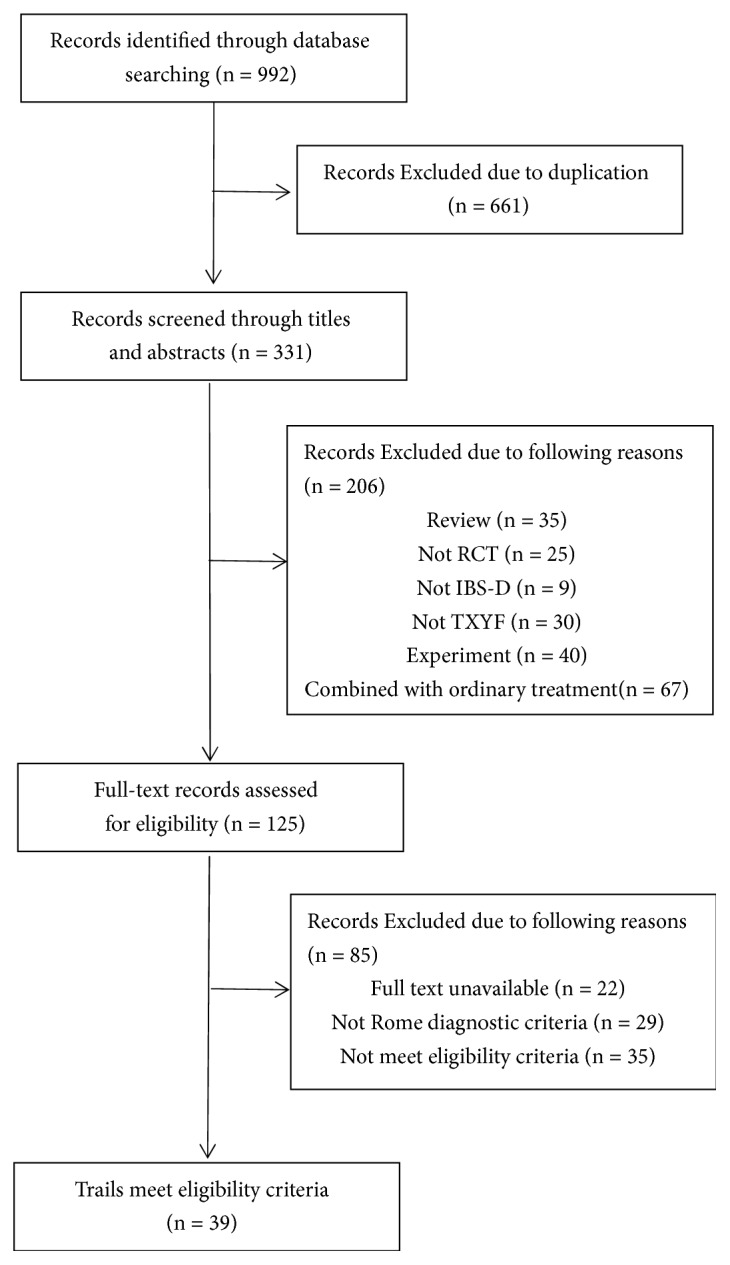


992 articles through electronic searching were identified. After duplications removed, 331 records were screened through titles and abstracts. Finally, 39 studies which met the inclusion criteria were included in this meta-analysis (Figure 1).

All trails are originated from China and published from 2006 to 2017 [8–46]. The total number of participants analyzed in the meta-analysis was 3062, of which 1556 received TXYF, while 1506 received ordinary treatment (Table 1).

### 3.2. Clinical Effectiveness Rate

**Figure 2:**
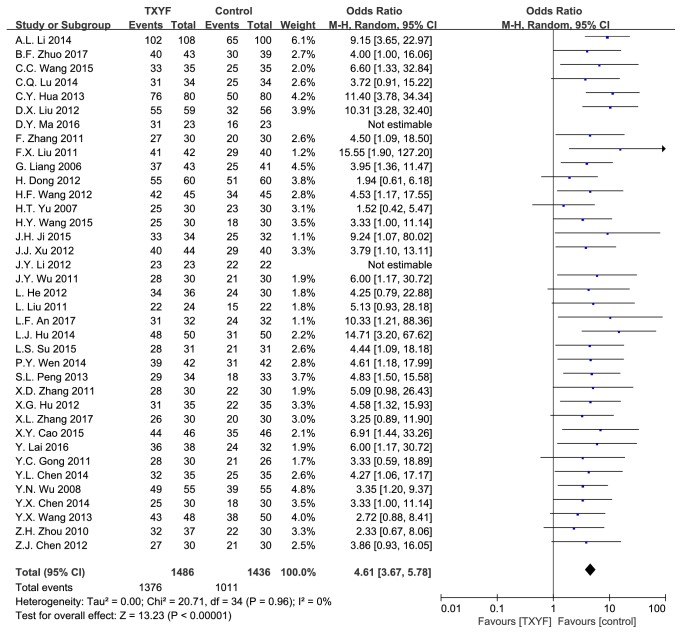


37 trials reported data on the clinical effectiveness rate. As shown in Figure 2, there was no significant heterogeneity (*I*^2^= 0%, P = 0.96). A random-effects model showed a significant improvement in the clinical effective rate (OR: 4.61; 95% CI: 3.67–5.78; P < 0.00001).

### 3.3. Abdominal Pain Score

**Figure 3:**
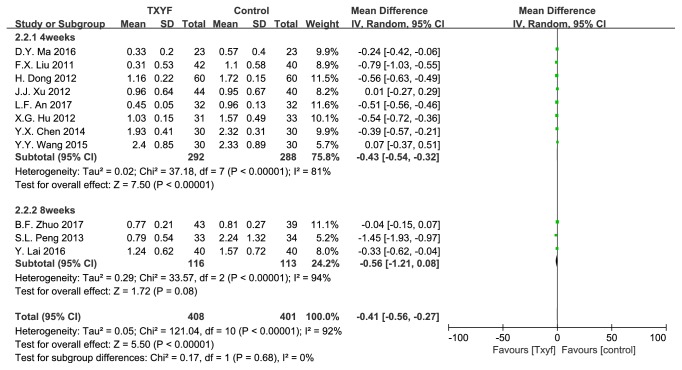


11 trails reported abdominal pain score. Trails were divided into two subgroups based on intervention duration (4w/8w). There was significant heterogeneity in test for overall and subgroup (Figure 3).

### 3.4. Defecating Frequency Score

**Figure 4:**
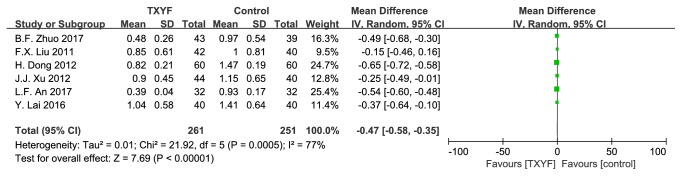


6 trails reported defecating frequency score. As shown in Figure 4, there was significant heterogeneity (*I*^2^ = 77%, P = 0.0005).

### 3.5. Fecal Property Score

**Figure 5:**
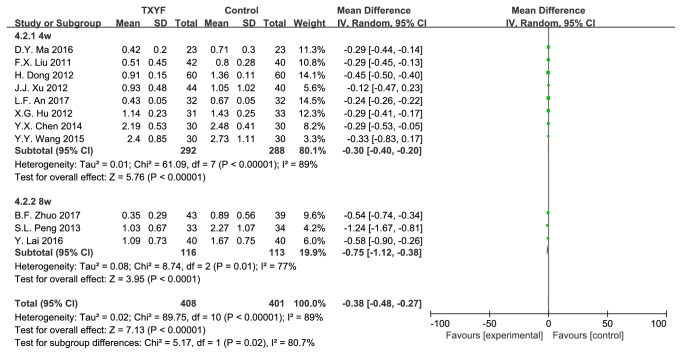


11 trails reported fecal property score. Trails were divided into two subgroups based on intervention duration (4w/8w). There was significant heterogeneity in test for overall and subgroup (Figure 5).

### 3.6. Total Symptom Score

**Figure 6:**
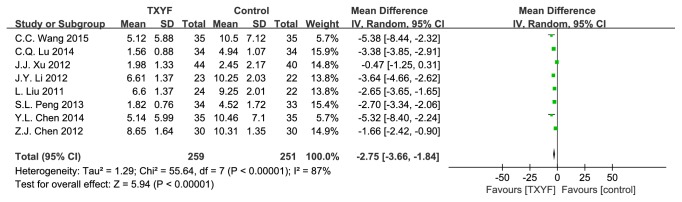


8 trails reported defecating frequency score. As shown in Figure 6, there was significant heterogeneity (*I*^2^ = 87%, P < 0.00001).

### 3.7. Adverse Effect Rate

**Figure 7:**
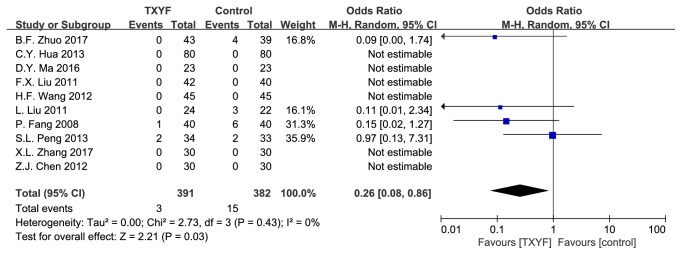


10 trails were involved.  Figure 7 showed that there was significant association with adverse effect rate (*I*^2^ = 0%, P = 0.43; OR: 0.26; 95% CI: 0.08–0.86; P = 0.03).

### 3.8. Recurrence Rate

**Figure 8:**
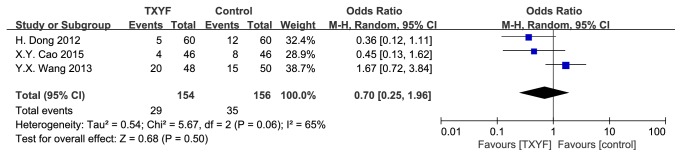


3 trails were involved. As shown in Figure 8, there was significant heterogeneity (*I*^2^ = 65%, P = 0.06).

### 3.9. Assessing Risk of Bias of Included Studies

11 trails were graded as low quality due to inappropriate randomization and/or allocation concealment. 9 trails were graded as high quality.

**Figure 9:**
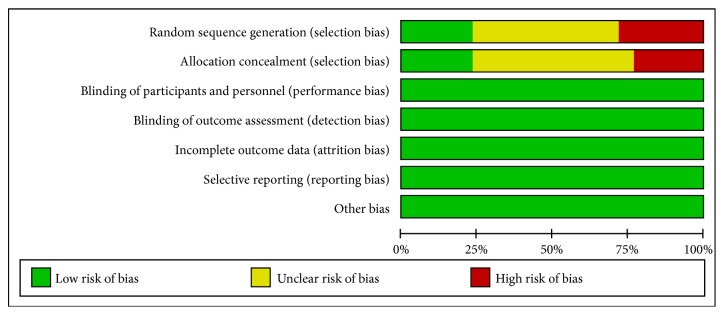


**Figure 10:**
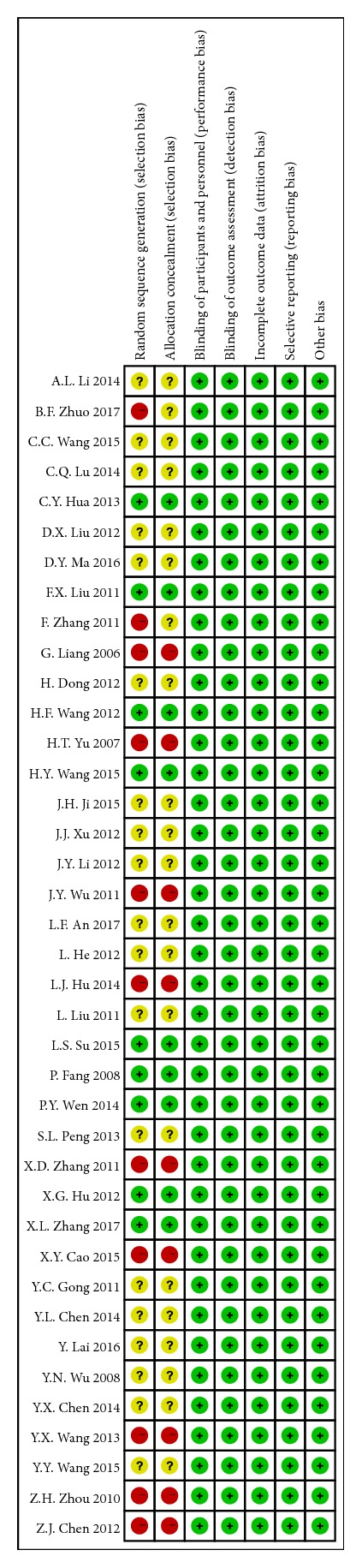


The risk of bias was moderate, shown in Figures 9 and 10 (+ indicated low risk of bias, - indicated high risk of bias, and ? indicated unclear risk of bias). 9 studies were given a low risk of random sequence generation due to the description the method of randomization namely random numbers table. Although double-blinded was not actualized in any trail included, the risk of blinding of participants and personnel and blinding of outcome assessment was low. Because, after discussion, we agreed that lack of blinding would not interfere the results seriously. Not any study included reported participants dropped out from any groups. So they were all assessed as low risk of bias of incomplete outcome data. All studies measured every anticipated outcome related to IBS-D mentioned before, so low risk of bias of selective outcome reporting was given to each study. The number of trials with low quality, high quality, and moderate quality was 9, 19, and 19, respectively. So we consider that the quality of identified studies was not high.

## 4. Discussion

To our knowledge, this was the first meta-analysis which critically evaluated the efficacy and safety of TXYF for IBS-D in English. The results in our study showed that, compared to conventional medication, TXYF appeared to be more effective in reducing adverse events rate (n=10, OR: 0.26; 95% CI: 0.08–0.86; P = 0.03) and improving the clinical effective rate (n=37, OR: 4.61; 95% CI: 3.67–5.78; P < 0.00001). There was not significant association with the score of abdominal pain, defecating frequency, fecal property, and total symptom comparing TXYF with conventional medicine. Probably, that was blamed to different evaluation criteria which were taken on clinical effectiveness and symptom score.

Conventional medication in our study, the positive control group, included probiotics, pinaverium bromide, trimebutine, and Oryzanol. The pathophysiology of TYXF treating IBS-D was not even understood. MiRNAs played a pivotal role in visceral hypersensitivity and might be targets in the treatment of IBS by Tongxieyaofang [47]. TXYF attenuated postinfectious IBS symptom by attenuating behavioral hyperalgesia and antidiarrhea, mediated by inhibiting PAR-2 receptor expression, reducing the levels of SP, TNF-*α*, and IL-6 in colonic mucosa, and decreasing fecal serine protease activity[48].

Traditional Chinese medicine was widely used as alternative and complementary medicine in China, Japan, and Korea. However, we could not find any studies originated from Japan or Korea from these databases mentioned above. All studies included were published in Chinese journals and conducted in China. It may be due to the authors of our study who all came from China.

In the inclusion criteria, we did not put any limits on TXYF combined with any other formulas or not, because TXYF and other formulas were part of TCM. Combination would not bring any risk of bias.

However, we should admit that several limitations concerning this study largely pertain to the incompleteness of the reported evidence. Firstly and foremost, the sample sizes of RCTs included were small and limited. Studies with small sample size, including publication bias, distorted the estimation of the effectiveness of an intervention under scrutiny in our review. It was difficult to find out the influence of contingency factors and increased the risk of bias. Secondly, the inadequate reporting on random sequence generation and none of the included trials which reported allocation concealment induced the selection bias occurring in the methodological designs of included studies, although our review processes were appraised rigorously by two experienced and independent authors. Thirdly, all trials included did not report double-blinding method, because we did not put any limits on types of intervention, so after discussion we agreed that lack of blinding would not bring the risk of bias and interfere in the results seriously. However, this opinion was conflicted with other meta-analyses [7].

Because the risk of bias of the finally included trails was not high, we suggested a moderate recommendation for TXYF on IBS-D. Considering that all identified studies were not high quality and large samples, further rigorously designed and large scale RCTs were necessary to improve the applicability of our study results.

## Figures and Tables

**Table 1:** The baseline characteristics of included trails.

Author	Year	Sample Size (I/C)	Control Group	Age (years)	IBS-D Duration	Intervention	Outcome Measurements
				(I/C)	(I/C)	Duration	
L.F. An	2017	32/32	Dioctahedral smectite+pinaverium bromide	34.8±3.8/34.2±3.6	3.06±0.34/2.97±0.32y	4w	①+②+③+④

X.Y. Cao	2015	46/46	Pinaverium bromide+oryzanol	18-56/16-58	2.5/2.2y	1m	①+⑦

Y.L. Chen	2014	35/35	Pinaverium bromide	40.23±14.85/38.57±12.49	NR	1m	①+⑤

Y.X. Chen	2014	30/30	Trimebutine+Bifid Triple Viable Capsules	43.1±10.2	NR	4w	①+②+④

Z.J. Chen	2012	30/30	Trimebutine	32.0±1.7/31.0±5.9	2.6±0.65/2.5±0.77y	30d	①+⑤+⑥

H. Dong	2012	60/60	Pinaverium bromide	26-57/28-53	2-11/1.5-10y	4w	①+②+③+④+⑦

P. Fang	2008	40/40	Pinaverium bromide+Bifid Triple Viable Capsules	41/42	3m-6y/2m-5y	1m	⑥

Y.C. Gong	2011	30/26	Bifidobacterium tetra viable tablets	33.3±12.3/32.6±11.1	3.3/3.8y	4w	①

L. He	2012	36/30	Bacillus subtilis and Enterococcus bacteria capsule	18-64/18-62	1.2-6/1.5-6y	4w	①

L.J. Hu	2014	50/50	Bifid Triple Viable Capsules	40.3±11.23/45.4±13.29	6m-8y/6m-7y	4w	①

X.G. Hu	2012	35/35	Pinaverium bromide	NR	NR	28d	①+②+④

C.Y. Hua	2013	80/80	Pinaverium bromide	17-27	NR	10d	①+⑥

J.H. Ji	2015	34/32	Montmorillonite+Bacillus subtilis and Enterococcus bacteria capsule	33.2±12.6/32.8±11.0	3.4±1.1/3.5±1.2y	5w	①

Y. Lai	2016	38/32	Bifid Triple Viable Capsules	67.4±13.6/64.5±12.8	3.9±1.5/3.8±1.6	8w	①+②+③+④

A.L. Li	2014	108/100	Combined Bifidobacterium and Lactobacillus Tablets	18-65	NR	8w	①

J.Y. Li	2012	23/22	Pinaverium bromide	42.56±10.71/41.38±11.25	15.72±10.35/15.48±10.52m	2w	①+⑤

G. Liang	2006	43/41	Pinaverium bromide	36.20±3.15/37.11±2.05	3.54±1.25/3.62±1.24y	4w	①

D.X. Liu	2012	59/56	Pinaverium bromide	45/44	1-6y/7m-7y	4w	①

F.X. Liu	2011	42/40	Loperamide	42±12/42±13	1-10/1-11y	4w	①+②+③+④+⑥

L. Liu	2011	24/22	Pinaverium bromide	43.55±13.79/38.70±10.76	6.30±5.52/5.61±5.51y	4w	①+⑤+⑥

C.Q. Lu	2014	34/34	Otilonium bromide	38.0/37.5	4.3/4.6y	4w	①+⑤

D.Y. Ma	2016	23/23	Pinaverium bromide	40/39	NR	4w	①+②+④+⑥

S.L. Peng	2013	34/33	Pinaverium bromide	40.3±11.9	NR	8w	①+②+④+⑤+⑥

L.S. Su	2015	31/31	Pinaverium bromide	35.6±3.4/34.5±3.7	2.9±1.2/2.7±1.5y	2w	①

H.F. Wang	2012	45/45	Dioctahedral smectite	45.2±12.5/43.2±11.7	35.5±12.3/36.7±13.5m	4w	①+⑥

H.Y. Wang	2015	30/30	Trimebutine+Bifid Triple Viable Capsules	41.4±11/42.5±10.6	NR	4w	①

Y.X. Wang	2013	48/50	Pinaverium bromide	27±4.5/29±5.1	3±2.7/3.3±2.4y	1m	①+⑦

Y.Y. Wang	2015	30/30	Pinaverium bromide+Bifid Triple Viable Capsules	46.8/48.9	3.7/3.4y	4w	②+④

C.C. Wang	2015	35/35	NR	40.56±15.02/39.54±13.23	6.80±4.30/8.20±4.69m	4w	①+⑤

P.Y. Wen	2014	42/42	Pinaverium bromide	41.7±11.6/42.4±12.3	36±12.5/37±13.1m	4w	①

J.Y. Wu	2011	30/30	Trimebutine+Bifid Triple Viable Capsules +Vitamin K	40.3±11.23/45.4±13.29	14m-8y/12m-7y	4w	①

Y.N. Wu	2008	55/55	Pinaverium bromide	18-60/16-61	1-30/1-28y	6w	①

J.J. Xu	2012	44/40	Pinaverium bromide	41.8±6.80/43.5±7.3	3.5/4.1y	28d	①+②+③+④+⑤

H.T. Yu	2007	30/30	Bifidobiogen	35.6/36.7	3.7/3.6y	30d	①

F. Zhang	2011	30/30	Dioctahedral smectite	18-69/19-68	1-24/2-23y	4w	①

X.L. Zhang	2017	30/30	Pinaverium bromide	36.7±9.5/36.1±8.2	4.2±1.1/4.5±1.4y	1m	①+⑥

X.D. Zhang	2011	30/30	Oryzanol+Dioctahedral smectite	NR	NR	4w	①

Z.H. Zhou	2010	37/30	Trimebutine	36.2/38.3	4.2/5.5y	14d	①

B.F. Zhuo	2017	43/39	Bifidobacterium tetra viable tablets+Pinaverium bromide	36.3±13.1/35.2±14.3	7.7±5.1/7.4±5.3y	8w	①+②+③+④+⑥

y, year; m, month; w, week; d, day; NR, not reported.

Note: ①the clinical effective rate, ②the score of abdominal pain, ③the score of defecating frequency, ④the score of fecal property, ⑤the score of total symptom, ⑥the adverse effect rate, and ⑦the recurrence rate.
